# Bone histology provides insights into the life history mechanisms underlying dwarfing in hipparionins

**DOI:** 10.1038/s41598-018-35347-x

**Published:** 2018-11-21

**Authors:** Guillem Orlandi-Oliveras, Carmen Nacarino-Meneses, George D. Koufos, Meike Köhler

**Affiliations:** 1 Institut Català de Paleontologia Miquel Crusafont (ICP), Campus de la Universitat Autònoma de Barcelona, 08193 Bellaterra, Barcelona Spain; 2 Department of Geology, Laboratory of Geology and Palaeontology, Aristotle University of Thessaloniki, 54124 Thessaloniki, Greece; 3 ICREA, Pg. Lluís Companys 23, 08010 Barcelona, Spain

**Keywords:** Evolutionary ecology, Palaeontology

## Abstract

Size shifts may be a by-product of alterations in life history traits driven by natural selection. Although this approach has been proposed for islands, it has not yet been explored in continental faunas. The trends towards size decrease experienced by some hipparionins constitute a good case study for the application of a life history framework to understand the size shifts on the continent. Here, we analysed bone microstructure to reconstruct the growth of some different-sized hipparionins from Greece and Spain. The two dwarfed lineages studied show different growth strategies. The Greek hipparions ceased growth early at a small size thus advancing maturity, whilst the slower-growing Spanish hipparion matured later at a small size. Based on predictive life history models, we suggest that high adult mortality was the likely selective force behind early maturity and associated size decrease in the Greek lineage. Conversely, we infer that resource limitation accompanied by high juvenile mortality triggered decrease in growth rate and a relative late maturity in the Spanish lineage. Our results provide evidence that different selective pressures can precipitate different changes in life history that lead to similar size shifts.

## Introduction

Body size is a key aspect of organisms’ biology as it is tightly correlated with many aspects of their physiology, ecology and life history^1–3^; body size, hence, is a fitness component^4^. Based on the coupling between life history traits and body mass, some authors^5–8^ have addressed size shifts in insular environments within the framework of life history theory.

Life history theory is built around the idea that selection acts on the life history characteristics of an organism to maximise its reproductive success or fitness^9,10^. To explain the range of life history strategies observed in nature, evolutionary biologists have traditionally used the approach of life history optimization^11^. Different optimality studies have provided a broad range of predictive models for the evolution of life cycle traits under different ecological constraints^9,12,13^. These models provide an explanatory framework for understanding how selective pressures can shape the life history of an organism.

Based on this scheme, Palkovacs^7^ proposed that body size shifts on islands can indirectly result from variation in life history traits instead of direct selection acting on size, as previously suggested by other authors^14–16^. He pointed out that two factors, extrinsic mortality and resource availability, influence life history traits to which adult size is sensitive, especially individual growth rate and age at maturity^7^. Although this approach has been theoretically proposed^7^ and tested^17–20^ in insular environments, it has not yet been explored in continental settings, where predictive life history models should also be applicable to explain body size evolution.

On continents, trends in size reduction have been identified in various taxonomic groups from different stratigraphic ages^21–23^. For instance, contrary to the notion of size increase over geological time within the horse lineage (Copes’ Rule)^24^, some equid clades experienced dwarfing^21,25,26^. This is the case of the *Arenahippus* lineage during the Paleocene-Eocene Thermal Maximum^27^, of some hipparionins during the European late Miocene^28–30^, and of the *Equus* lineage during the Pleistocene^25,31,32^.

The first hipparionins that dispersed throughout the Old World in the Vallesian were large sized hipparions^33^ that likely evolved from a large ancestor^34^. Late Miocene hipparionins from the Eastern and Western Mediterranean basins exemplify an interesting case of continental trends in size decrease. The Western Mediterranean late Turolian (MN13) dwarf hipparions from the Teruel Basin (Spain) have been interpreted to represent an evolutionary gradation towards reduced body size, simplified enamel abrasive figures, increasing hypsodonty and gracility^29,30^. This size reduction led to some of the smallest *Hipparion sensu lato* forms of the Old World, *Hipparion gromovae* and *Hipparion periafricanum*, with estimated mean body masses of 59 kg and 23 kg respectively^29^. These two taxa coexisted sympatrically with the larger 138 kg *Hipparion truyolsi*^29^. In the Eastern Mediterranean basins, the existence of the small *macedonicum* morphotype is already reported in the early Vallesian (MN9), and its temporal range extends up to the middle Turolian (MN12), and possibly to the late Turolian (MN13)^28,35^. Similar to the Spanish small hipparionins, the Greek *Hipparion macedonicum* underwent a body mass decrease coupled with a simplification of the enamel plication and an increased gracility of the metapodials^28^. The body mass estimations for this taxon range from 72.3–121 kg during the Vallesian (MN9-MN10) to 49.5–94.7 kg during the early-middle Turolian (MN11-12)^28^. This small morphotype coexisted with larger species that pertain to the *primigenium*, the *dietrichi* and the *proboscideum* morphotypes, which almost double the body mass of the small forms^35^.

The occurrence of these dwarfed hipparionins in the European late Miocene represents a good opportunity to test for the coupling of life history changes and size shifts. In the present study, we aim to unravel the possible mechanisms and causes underlying trends in size reduction of these hipparions from a life history approach. For this purpose, we reconstruct the bone growth pattern of different-sized hipparionins through bone histology. Due to the fact that tissue type and bone growth marks reflect tempo and rate of bone growth^36,37^, bone histology is a valuable tool for reconstructions of life histories^38,39^ and growth patterns^40–42^.

## Results

### Bone histology

Bone microstructure is generally better preserved in the Spanish specimens (Supplementary Fig. S1a), while some Greek metapodials have suffered different taphonomical alterations. In most cases this does not erase histological details such as vascularization or bone growth marks (Supplementary Fig. S1b). The sample from the Nikiti-2 (NIK) fossil site, however, is especially damaged diagenetically or through microbiological attack (Supplementary Fig. S1c), and it has therefore not been used for our growth curves reconstruction.

**Figure 1:**
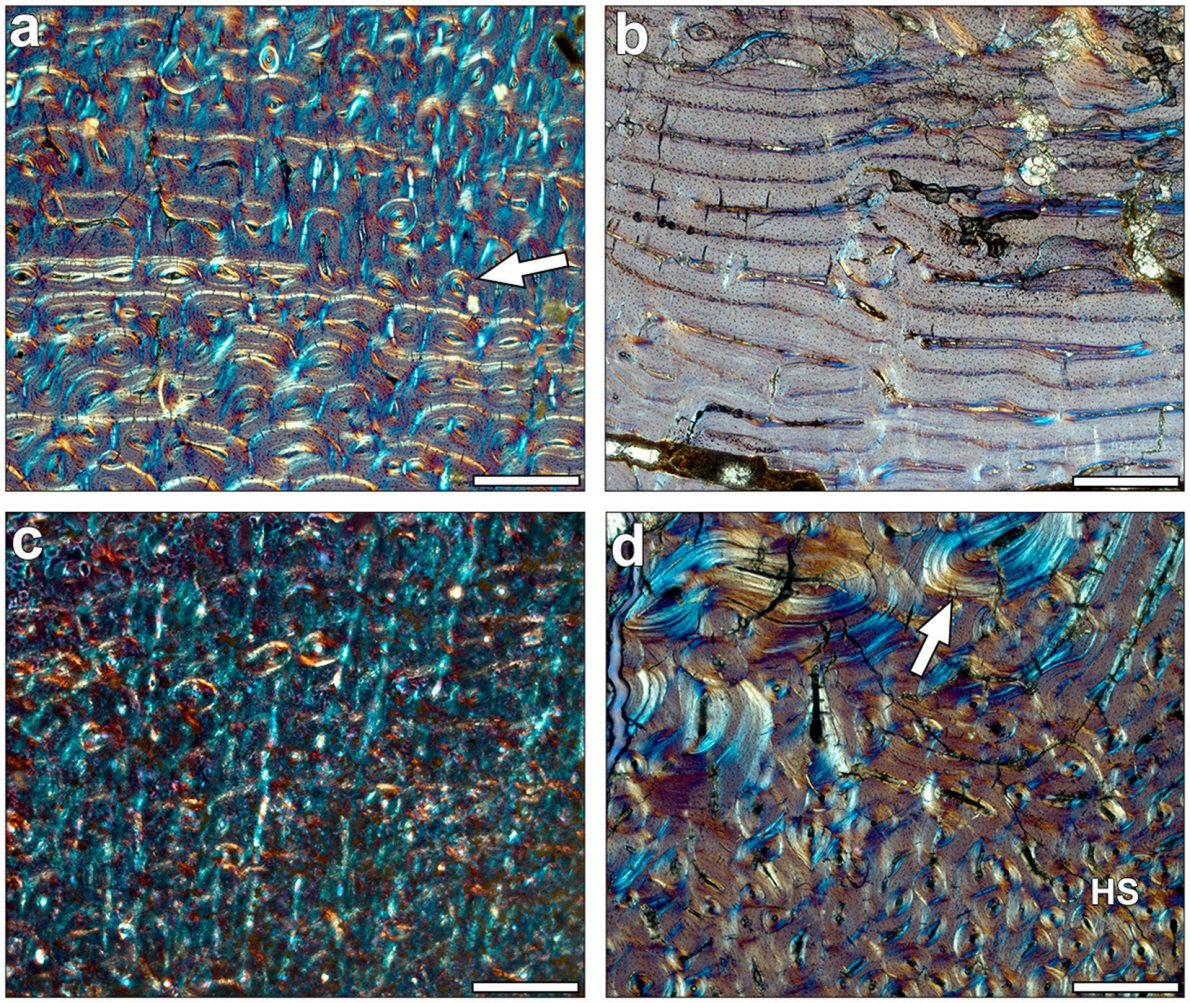
Hipparionin metapodial histology observed under polarised light using a 1/4λ filter. Scale: 0.5 mm. (**a**) Primary bone tissue formed by fibrolamellar complex (FLC) in the innermost cortex of the *Hipparion truyolsi* metatarsal IPS-28842. The arrow points out the transition between a FLC with higher parallel fibered bone component (PFB) to a FLC with higher fibrous component and larger osteons. (**b**) Laminar bone tissue in the inner cortex of the *Hipparion* cf. *sebastopolitanum* (*primigenium* morphotype) PNT-22. (**c**) Radial vascular canals present in the first growth cycle of the *Hipparion philippus* (*dietrichi* morphotype) metatarsal PER-342. (**d**) Secondary bone tissue in the lateral posterior area of the *Hipparion gromovae* metacarpal IPS-101807. Dense Haversian systems (HS) are concentrated in the contact zone with the lateral metapodials, and lamellar tissue is filling the resorption cavities near the medullary area (arrow indicating resorption line).

Both metacarpals and metatarsals of all the groups studied present a similar tissue and vascular arrangement. Their primary bone consists of a fibrolamellar complex (FLC) with longitudinal primary osteons oriented in circular rows (Fig. 1a). A similar histological pattern has been observed in previous studies in the metapodials of extant^43^ and fossil equids^44,45^. The amount of parallel fibered bone (PFB) within the FLC^46^ is higher in the innermost cortex of the metapodials than in the outer cortical area, coinciding with a smaller diameter of the primary osteons in the inner cortex (Fig. 1a). This observation is in agreement with the tissue pattern observed in extant equid bones^43,47^.

We also have identified the following primary tissues and vascular orientations within the histological samples: Laminar bone is present in the posterior inner cortical zone (Fig. 1b) in some metapodials. Half of the Spanish *H. gromovae* specimens exhibit this tissue in the innermost cortex, while it is present in only some metapodials from Greek Vallesian sites. Radial canals are also identified within the first growth cycle of metatarsals of the large *dietrichi* morphotype (Fig. 1c) and one *primigenium* metatarsal (PNT-4). Radial canals are also sparsely situated in some regions of one *dietrichi* metacarpal (DTK-58) and in metapodials of the *macedonicum* morphotype (PER-23 and PER-425). The different vascular arrangements exhibit modest differences in growth rate compared to the bone matrix typology^48^. Nevertheless, tissues with radial osteons present higher growth rates compared to those with predominant circular osteons, as laminar tissues, when observed within the same bone^49,50^. Accordingly, the radial canals mainly observed in some *dietrichi* and *primigenium* metatarsals can be related to higher growth rates, while the laminar bone tissue present in four of the eight *H. gromovae* specimens can be an indicator of slower growth rates. These extrapolations from bone vascularization patterns must be done carefully in fossil taxa because of the high range of variability^48,49^.

**Figure 2:**
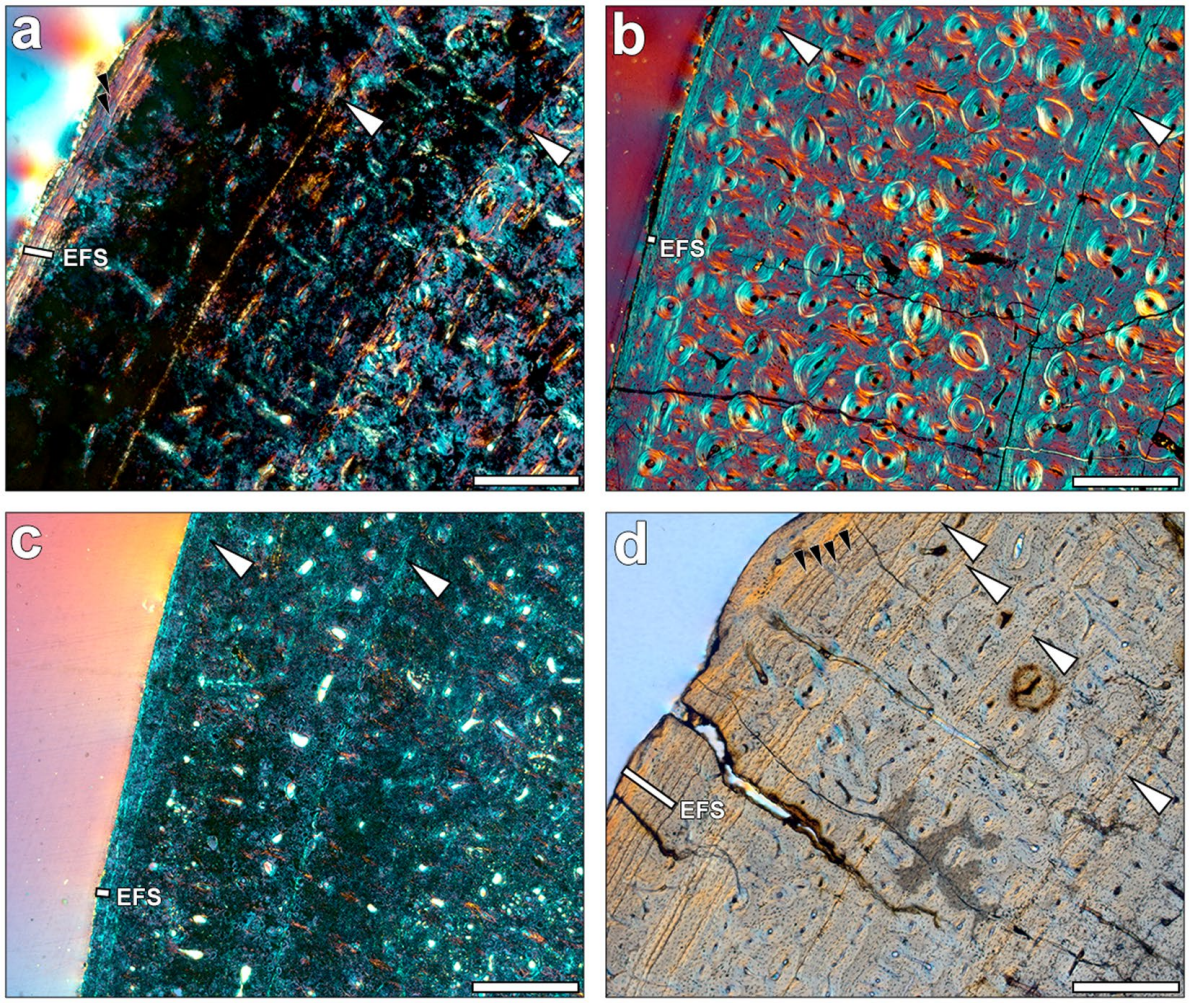
Bone growth marks in the periosteum of adult metacarpals. Growth marks are indicated by white arrows, small black arrows mark the growth lines present within the EFS, which is identified by a white line. The images were obtained under polarised light using a 1/4λ filter. Scale: 0.5 mm. (**a**) *Hipparion* aff*. giganteum* (*primigenium* morphotype) metacarpal NKT-22. (**b**) *H. philippus* (*dietrichi* morphotype) metacarpal PER-X. (**c**) *Hipparion macedonicum* (*macedonicum* morphotype) metacarpal PER-23. (**d**) *H. gromovae* metacarpal IPS-96274.

**Figure 3:**
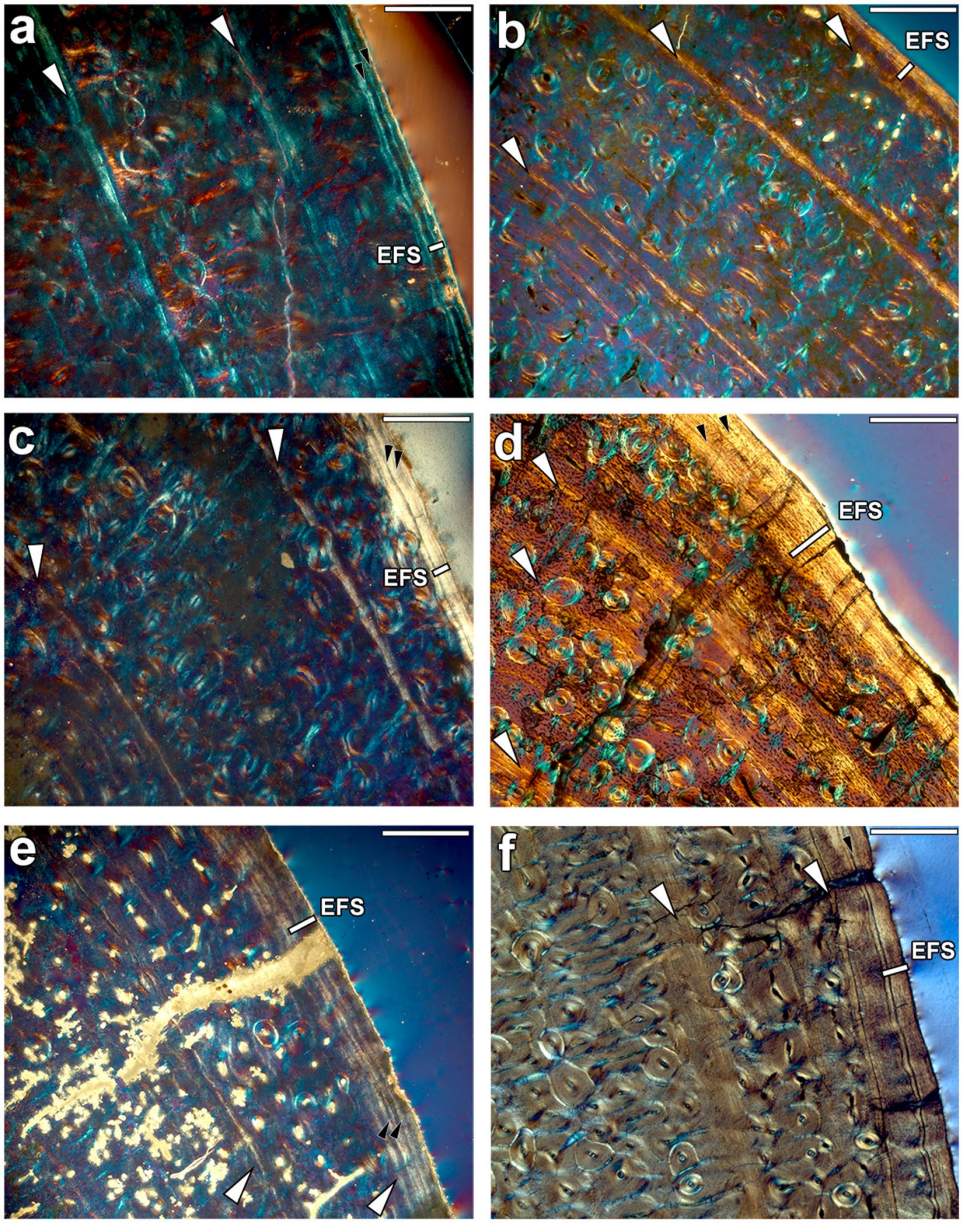
Bone growth marks in the periosteum of adult metatarsals. Growth marks are indicated by white arrows, small black arrows mark the growth lines present within the EFS, and EFS is identified by a white line. The images were obtained under polarised light using a 1/4λ filter. Scale: 0.5 mm. (**a**) *H*. cf. *sebastopolitanum* (*primigenium* morphotype) metatarsal PNT-4. (**b**) *Hipparion* aff.* platygenys* (*dietrichi* morphotype) metatarsal DTK-106. (**c**) *H. philippus* (*dietrich*i morphotype) metatarsal PER-1211. (**d**) *H. gromovae* metatarsal IPS-101809. (**e**) *H. macedonicum* (*macedonicum* morphotype) metatarsal PER-485. (**f**) *H. truyolsi* metatarsal IPS-28842.

We identified large resorption cavities (RC) distributed near the medullary cavity within the posterior area of almost all metapodials. These RC appear early in ontogeny and, in some cases, lamellar bone is deposited at their margins, resulting in a high concentration of secondary tissue in the inner posterior zone of the metapodials (Fig. 1d). Besides, dense Haversian tissue is identified in our samples (Fig. 1d). The Haversian tissue is mainly concentrated within the lateral posterior and medial posterior areas, where the lateral metapodials (II and IV) contact the central one. The major secondary remodelling of these areas is related to higher biomechanical loadings^43,44^. Two metapodials show an extensive distribution of Haversian systems (PER-X and IPS-28842), though the growth marks are still identifiable (Supplementary Fig. S2). Superimposition of different ontogenetic stages shows that neither medullary expansion nor Haversian canals completely eroded the cyclical growth marks (CGMs). Lamellar bone is present in the outermost cortex of some metapodials, forming the external fundamental system (EFS, see Material and Methods) in adult individuals that have attained their final size (Figs 2 and 3).

### Skeletocronology

We have used the specimens that approached their final size for skeletocronological analysis and growth reconstruction. In most cases, the three Greek morphotypes present two CGMs embedded in the FLC matrix, both in the metacarpals (Fig. 2a,b) and the metatarsals (Fig. 3a,b,e). However, one of the three metatarsals of the *dietrichi* morphotype (DTK-106) exhibits three CGMs in the FLC prior to deposition of the EFS in the lateral and medial areas, while two of the five specimens of the *macedonicum* morphotype (PER-23 and VAT-112) have only one CGM each. This can suggest an advanced maturity in the small Greek representatives though there appears to be some variability. The main difference between the Greek forms, however, lies in the spacing between the last growth mark of the FLC and the EFS. Generally, when analogous regions are compared, the large *primigenium* and *dietrichi* metacarpals (Fig. 2a,b) continue to grow after the second CGM compared to the *macedonicum* morphotype (Fig. 2c). Similarly, a higher amount of tissue is deposited during the third growth cycle of the larger metatarsals of *primigenium* and *dietrichi* morphs (Fig. 3a–c), while the small *macedonicum* metatarsals deposits fewer tissue in this zone (Fig. 3e). This early narrowing of the growth zones^51^ lends support to the idea of an earlier attainment of maturity in the smaller Greek form compared to the larger ones. Within the Spanish hipparionins, the *H. gromovae* metapodials show three CGMs in the bone cortex prior to the EFS (Figs 2d and 3d), while the larger *H. truyolsi* metatarsal has only two CGMs embedded in the FLC (Fig. 3f). Although it has not reached maturity, the *H. gromovae* metacarpal IPS-96275 (Supplementary Fig. S1a) also points towards extended growth period as it exhibits open vascular canals in the periosteum after the deposition of the second CGM. Consequently, the metapodials of the small *H. gromovae* present three or more cycles of active growth, contrary to the small Greek morphotype and the larger Spanish taxon. These differences in growth cessation can also be identified by the overall growth trajectory reconstruction.

### Growth Curves Reconstruction

**Figure 4:**
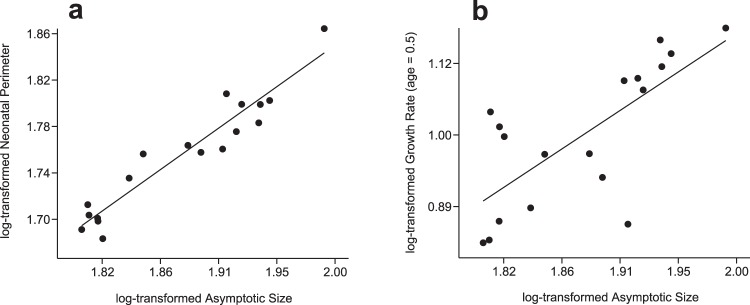
(**a**) Linear regression between the neonatal metapodial size and the adult final size (*A*) of mature metapodials. (**b**) Allometric regression relating the growth rate during the first growth cycle (t = 0.5 years) and the adult final body size (*A*) of mature metapodials. All variables were log-transformed to linearise the relationship. See main text for statistics information.

The curve fitting allowed us to obtain characteristic growth parameters and to estimate growth variables (Table 1, see Material and Methods). At birth, metapodial size shows an almost isometric relationship (0.834-slope) with the asymptotic maximum size obtained for each specimen (parameter *A*) (R^2^ = 0.908; p-value (Pearson) < 0.001) (Fig. 4a). Therefore, metapodial postnatal growth starts at analogous sizes in each hipparionin group, depending on their adult final circumference. If we consider bone perimeter as a proxy of body mass^50,52^, these values correspond to those predicted from isometric scaling of eutherian neonatal mass with adult body mass^1^. This contrasts with insular dwarf lineages where neonate size is smaller than predicted from allometry^18^. After birth, the growth rate during the first growth cycle (age = 0.5 years, Table 1) also scales positively with adult bone size (Fig. 4b, R^2^ = 0.543; p-value (Pearson) < 0.001). However, when the same relationship is established but considering the relative growth rate at that point (size factor extracted), the correlation is not significant (R^2^ = 0.160; p-value (Pearson) > 0.05). During the first stages of growth, hence, metapodials grow at analogous velocities relative to their size, and the allometrical relationship with a 1.46 slope results in slightly higher growth rates in the metapodials of large forms compared to the small ones. For comparison, we have also related the growth rate and the femoral lateromedial diameter, a good proxy of body size in equids^53^, on a sample of different-sized extant equid femurs (see Material and Methods). Although the points of the obtained regression are scattered (R^2^ = 0.387) (Supplementary Fig. S3) and the correlation is not statistically significant (p-value (Pearson) = 0.099), we can identify a trend indicating higher growth rates in larger taxa and lower growth rates in the smaller species or breeds. Similar to what we observe in hipparionins, hence, extant smaller equid species and horse breeds, such as *Equus hemionus* and the Shetland pony, tend to grow at absolute slower rates than larger representatives of the genus *Equus*.

**Table 1:** Logistic growth curve parameters and growth estimates for each adult metapodial.

GROUP	BONE	CODE	A (mm)	k (year^−1^)	t95A (year)	GR (t = 0.5) (mm/year)	RGR (t = 0.5) (year^−1^)	GR (t = 1) (mm/year)	RGR (t = 1) (year^−1^)	GR (t = 2) (mm/year)	RGR (t = 2) (year^−1^)	GR (t = 3) (mm/year)	RGR (t = 3) (year^−1^)
*primigenium* morphotype	MC	NKT-22	81.54	0.66	2.46	7.35	0.108	5.79	0.081	3.36	0.044	1.85	0.023
	MC	RPL-nn	83.74	1.02	1.81	11.89	0.170	8.20	0.110	3.41	0.043	1.30	0.016
	MT	PNT-4	86.53	0.99	2.00	13.07	0.186	9.29	0.123	4.07	0.049	1.61	0.019
	**Mean**	***pgMC***	**82**.**64**	**0**.**84**	**2**.**07**	**9**.**60**	**0**.**139**	**7**.**08**	**0**.**097**	**3**.**49**	**0**.**045**	**1**.**60**	**0**.**020**
		***pgMT***	**86**.**53**	**0**.**99**	**2**.**00**	**13**.**07**	**0**.**186**	**9**.**29**	**0**.**123**	**4**.**07**	**0**.**049**	**1**.**61**	**0**.**019**
*macedonicum* morphotype	MC	RPL-44	69.06	0.86	1.94	7.75	0.133	5.63	0.091	2.69	0.041	1.20	0.018
	MC	PER-23	65.57	1.27	1.42	10.31	0.184	6.30	0.105	2.00	0.031	0.59	0.009
	MC	PER-425	64.47	0.88	1.83	6.99	0.127	5.01	0.086	2.35	0.038	1.03	0.016
	MT	VAT-112	64.60	1.88	0.90	10.91	0.188	4.83	0.078	0.79	0.012	0.12	0.002
	MT	PER-485	70.71	1.32	1.16	9.32	0.149	5.38	0.081	1.57	0.023	0.43	0.006
	**Mean**	***mcMC***	**66**.**37**	**1**.**00**	**1**.**69**	**8**.**40**	**0**.**149**	**5**.**74**	**0**.**096**	**2**.**39**	**0**.**037**	**0**.**92**	**0**.**014**
		***mcMT***	**67**.**66**	**1**.**60**	**1**.**01**	**10**.**28**	**0**.**170**	**5**.**21**	**0**.**081**	**1**.**14**	**0**.**017**	**0**.**23**	**0**.**003**
*dietrichi* morphotype	MC	PER-X	78.02	0.65	3.05	8.59	0.141	7.03	0.108	4.33	0.061	2.48	0.034
	MC	DTK-58	76.29	0.85	2.14	9.34	0.148	6.92	0.103	3.42	0.048	1.56	0.021
	MT	PER-1211	82.96	0.94	2.16	12.46	0.188	9.11	0.127	4.23	0.054	1.77	0.022
	MT	PER-342	86.29	1.04	2.02	14.63	0.213	10.32	0.138	4.35	0.053	1.64	0.019
	MT	DTK-106	87.97	1.02	1.97	13.80	0.194	9.71	0.126	4.13	0.049	1.59	0.018
	**Mean**	***dtMC***	**77**.**15**	**0**.**75**	**2**.**54**	**9**.**08**	**0**.**146**	**7**.**08**	**0**.**107**	**3**.**92**	**0**.**055**	**2**.**00**	**0**.**027**
		***dtMT***	**85**.**74**	**1**.**00**	**2**.**04**	**13**.**63**	**0**.**198**	**9**.**72**	**0**.**130**	**4**.**24**	**0**.**052**	**1**.**67**	**0**.**020**
*H*. *gromovae*	MC	IPS-101807	65.53	0.79	2.25	7.42	0.137	5.62	0.098	2.93	0.048	1.42	0.022
	MC	IPS-96274	63.83	0.77	2.29	6.93	0.131	5.29	0.095	2.82	0.047	1.40	0.023
	MT	IPS-101809	81.00	0.95	2.15	12.34	0.191	9.00	0.129	4.15	0.054	1.73	0.022
	MT	IPS-96276	66.09	1.01	1.92	9.94	0.184	6.96	0.119	2.96	0.047	1.14	0.018
	**Mean**	***grMC***	**64**.**68**	**0**.**78**	**2**.**27**	**7**.**17**	**0**.**134**	**5**.**45**	**0**.**096**	**2**.**88**	**0**.**047**	**1**.**41**	**0**.**023**
		***grMT***	**73**.**55**	**0**.**98**	**2**.**04**	**11**.**15**	**0**.**188**	**7**.**97**	**0**.**124**	**3**.**53**	**0**.**051**	**1**.**42**	**0**.**020**
*H*. *truyolsi*	MT	IPS-28842	97.00	1.30	1.39	15.41	0.185	9.27	0.104	2.85	0.030	0.80	0.008
	**Mean**	***tyMT***	**97**.**00**	**1**.**30**	**1**.**39**	**15**.**41**	**0**.**185**	**9**.**27**	**0**.**104**	**2**.**85**	**0**.**030**	**0**.**80**	**0**.**008**

Mean growth curve parameters and estimates for the metacarpals (MC) and metatarsals (MT) of each group are also provided. *A:* asymptotic circumferential metapodial size; *k*: mean relative velocity; *t95A*: time required to attain the 95% of the final size. Growth rates (GR) and relative growth rates (RGR) at different points of the metapodial growth are also shown.

**Figure 5:**
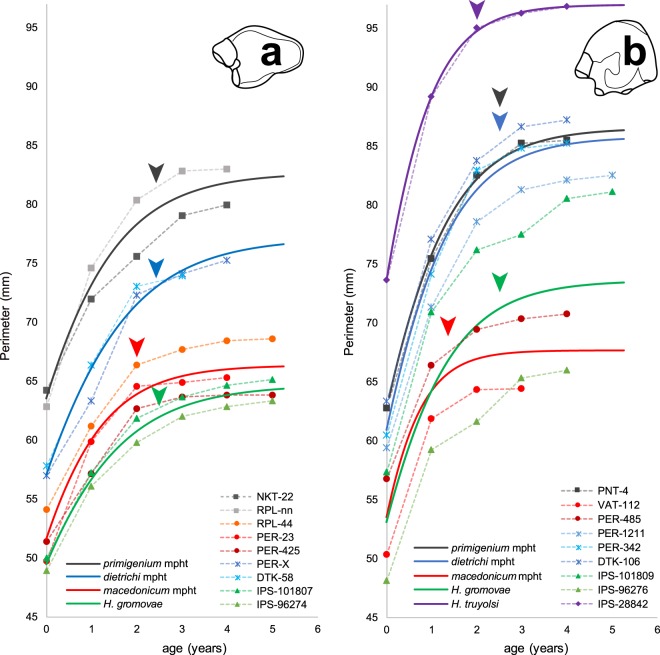
Growth curves of mature metapodials. Dashed lines represent each metapodial growth trajectory while solid lines indicate the mean fitted logistic growth curves for the different groups. Arrows pointed out the change of slope of the growth curves that characterises metapodial maturity^43^. (**a**) Metacarpal growth curves. (**b**) Metatarsal growth curves. The unusual size of the *H. gromovae* metatarsal IPS-101809 is due to a more proximal cut of the specimen, however, the growth trajectory is still identifiable.

The overall growth patterns in hipparionins are graphically visualised in the curves from discrete measurements of the CGMs (Fig. 5, dashed lines). From the obtained logistic parameters (Table 1), we reconstructed the mean growth trajectory for each group (Table 1 and Fig. 5, solid lines). In the mean and individual growth curves, we identified the point where the growth rate is reduced as the change in the slope of the trajectory^43^ (Fig. 5, arrow), which coincides with the lower bone deposition observed histologically. This signal has been related to epiphyseal fusion and reflects the end of longitudinal bone growth^43^. The growth rate of the larger Greek metapodials substantially declines by around ~2.5 years after birth, and generally at sizes above 75 mm of perimeter (Fig. 5). Growth deceleration starts earlier in the small Greek *macedonicum* morphotype, about the first year in the metatarsals and within the second year in the metacarpals, both at perimeter sizes around ~65 mm. The Spanish dwarf form, on the contrary, grows at sustained rates over a longer period until the age of approximately ~2.5 years (Fig. 5), comparable to the large Greek morphs. Indeed, the growth in *H. gromovae* metapodials and the larger Greek morphs declines later compared to the small Greek morphotype and to the larger Spanish *H. truyolsi* (Fig. 5), in which the absolute (GR) and relative (RGR) growth rates are considerably low at the second and third year of growth (Table 1 and Supplementary Fig. S4).

The logistic growth parameters (Table 1) also show the aforementioned growth differences. Accordingly, the growth parameter *k*, that represents the relative growth velocity (see Material and Methods), tends to be higher in the metapodials of the *macedonicum* morphotype (k_MC*mc*_ = 1 in metacarpals and k_MT*mc*_ = 1.6 in metatarsals, Table 1) than in the specimens of the other groups (e.g. *dietrichi* morphotype metacarpals k_MC*dt*_ = 0.84 and metatarsals k_MT*dt*_ = 1, Table 1), when metacarpals and metatarsals are compared separately. This indicates an initially faster growth relative to size, and an earlier attainment of maturity in this Greek dwarf form. In contrast, the small metapodials from *H. gromovae* and the larger from the large Greek morphotypes (*primigenium* and *dietrichi*) exhibit lower *k* values (e.g. k_MC*gr*_ = 0.78 and k_MT*gr*_ = 0.98, Table 1), which indicates a longer growth period and a later onset of maturity. Conversely, the large Spanish *H. truyolsi* has one of the highest *k* value (k_MT*ty*_ = 1.3, Table 1) as it grows fast and decreases the deposition rate before the second year of life (Fig. 5). The growth period, or the time required to attain the 95% of its final size (*t95A*), illustrates the same fast development in the *macedonicum* morphotype compared to the Spanish small species and the larger Greek morphs (Table 1). For instance, while the metatarsals of the *macedonicum* morphotype complete the 95% of growth by the age of 1 year (t95A_MT*mc*_ = 1.01, Table 1), the small metatarsals of *H. gromovae* and the larger ones from *dietrichi* and *primigenium* morphotypes double this time (t95A_MT*gr*_ = 2.04, t95A_MT*dt*_ = 2.04, t95A_MT*pg*_ = 2, Table 1). The single metatarsal of the larger *H. truyolsi* (t95A_MT*ty*_ = 1.39, Table 1) ceases growth earlier than the metatarsals of the other taxa except for the *macedonicum* morphotype. This earlier maturity in *H. truyolsi*, however, should be taken with caution since there is only one remodelled metatarsal available and there is no other metatarsal of this species to compare with. In all groups analysed, the metacarpals attain their final size later than metatarsals, as shown both by the parameter *k* and by the time required to complete growth (Table 1). This finding can be related to the variability reported between other limb bones of equids^43^ and the ontogenetic differences between bones^54^. Distinct loading charges and biomechanics between hipparion metapodials may be the reason underlying this difference^55^.

## Discussion

Body size variations in continental settings, and concretely in equid lineages, have been hypothesised to represent direct adaptations to habitat change^25,29^, resources shifts^27,30,31,34^ or to climate change^25,27^. These hypotheses are closely linked because of the strong relationship between climate, habitat and resources. Alternatively, life history theory provides a theoretical background to explain size shifts as a by-product of life history adaptations to environmental conditions. This approach has been proposed for islands^7^; however, it has not been explored in mainland size trends. Our analysis of the growth strategies of different-sized hipparionins provides a first attempt to understanding the possible causes behind continental trends in size decrease within a life history framework.

In the present study, we inferred the growth trajectories of small and large hipparionin groups from two continental bioprovinces using metapodial bone histology. Although the bone microstructure of metapodials does not provide the absolute age of physiological maturity^43^, it informs about the relative maturity that, within the context of body mass, indicates whether these size reductions were coupled with shifts towards either slower or faster life histories. Future histological analysis on adult femurs could provide an absolute estimation of the age at sexual maturity and, thus, help to support our inferences.

We found that the metapodials of the larger hipparionins grow at higher rates than the metapodials of the smaller forms during the first growth stages (Fig. 4b and Table 1). We can infer, hence, lower growth rates for the small taxa and higher rates for the larger forms as predicted from allometry^1^ and observed in dinosaurs^36,56^ and in extant and fossil artiodactyls^6,18,39,57^. Moreover, we identified a similar trend in extant equids, since femora of larger wild equids and of larger domestic breeds tend to grow at higher rates than the smaller-sized species or breeds (Supplementary Fig. S3). In hipparionins, although the differences in development have principally been identified from growth curve reconstructions, bone tissue microstructure also indicates distinct growth velocities. Thus, we principally found radial vascularity in the large *dietrichi* and *primigenium* metatarsals, and laminar bone in the small *H. gromovae*. In fact, growth rate is particularly low in *H. gromovae*, where besides the growth rate scaling (Fig. 4b), the relative growth rate expressed by the logistic growth parameter *k* is also lower (Table 1). On the other hand, the small *macedonicum* morphotype metapodials exhibit in some cases disperse radial canals, indicating a probable higher growth rate for its size, which is supported by the greater *k* values. Regarding the attainment of relative maturity, the small *macedonicum* morphotype and the large *H. truyolsi* considerably decrease their growth rate between the first and the second year, while the two large Greek morphotypes and the small Spanish *H. gromovae* decrease growth rate after the second year. The large Greek forms, hence, grew at high rates and attained their maturity later than the small Greek *macedonicum* morphotype that advance the growth rate decline. The small Greek *macedonicum* morphotype, thus, shifted towards a faster life history. The small Spanish *H. gromovae*, on the contrary, matured relatively late, similar to the large Greek representatives. However, it does it at much lower growth rates. *H. truyolsi*, on the other hand, grew at high rates and decreased growth early (based on a single specimen). Martinez-Maza *et al*.^44^ observed two CGMs within the cortex of the metapodials of the larger (160–186 kg^58^) *Hipparion concudense* from a Vallesian and a middle Turolian Spanish locality. These growth marks are situated close to the periosteal surface^44^, suggesting an early decrease in growth rate. Moreover, in half of the metapodials of their middle Turolian sample, only one CGM is situated within the bone cortex^44^. From allometric scaling^1^, the smaller *H. gromovae* should be expected to show less CGMs within the cortex than *H. truyolsi* or *H. concudense*; however, it exhibits more growth marks indicating a delayed maturity in relation to these larger Spanish species. Besides, our results have also shown that in some cases metacarpals tend to decrease their growth later than metatarsals. This difference is beyond the scope of the present study, but may indicate different timings in the growth cessation between these bones.

In summary, we did not only find differences in the growth patterns between the small and the large Greek morphotypes as predicted from scaling, but also between the small Greek *macedonicum* morphotype and the small Spanish *H. gromovae*, since the Spanish species matured later than expected from size. The two dwarf lineages, hence, followed different growth strategies that led to a similar reduction in adult size. Our results, thus, are congruent with two different evolutionary scenarios where dwarfing is caused by distinct selective pressures. Following life history theory, attainment of sexual maturity and growth rate are the two main characteristics that determine the final size of an organism^7,9^. These factors, in turn, are modulated by environmental changes in mortality regimes and resource availability respectively^7,9,12,59–61^. Palkovacs^7^, in his life history based model for size evolution on islands, considered the changes in resource levels and mortality rates (caused by low predation) between mainland and islands as the principal selective pressures involved in size shifts. However, variations in ecological factors over time and space can be far more complex on the continent where, for example, predation pressure can either increase or decrease, and can affect specific ontogenetic stages differently. As age-specific survival and fecundity indexes are the basis of life history and demographic studies^9,10,60^, it is important to determine which is the life cycle stage affected by a shift in the selective agent. Organisms that suffer from high extrinsic mortality regimes acting on *adult* stages will maximise their fitness following a different life history strategy compared to those exposed to high *juvenile* mortality^61,62^. Concretely, when the *adult* stage is affected by high predation pressure, animals maximise their fitness by reproducing earlier^62^. By this, organisms are able to increase their reproductive output before they fall prey to carnivores, hence accelerating their life history. Both theoretical^60,62^ and comparative surveys^59,61,63^ provide evidence for advancement of reproductive maturity in populations facing high adult mortality regimes. This suggests that increased predation pressure upon adult individuals selected for advanced maturity through earlier growth cessation, leading to dwarfing in the *macedonicum* lineage. On the other hand, under high *juvenile* mortality, organisms maximise fitness by reproducing later in time^12,13,60,61^. A delay in maturity is predicted if the benefits outweigh the disadvantages of a late reproduction^12,64^. These advantages are based on two key assumptions; fecundity increases with size, and juvenile mortality decreases with increasing age and size at maturity of the progenitor^12^. Because equids are monotocous^65^, a significant increase in fecundity related to larger size is not expected; however, a delay in maturity can compensate high juvenile mortality by increasing juvenile survival rates due to more experienced progenitors^59^. Age-specific juvenile mortality increases during periods of low resource availability^59,66–70^, which are common in arid and semi-arid environments^71^. Seasonal or unpredictable fluctuations in food quantity and quality have a high influence on juvenile mortality^72^, since at this ontogenetic stage young mammals are more sensitive to environmental stresses due to their smaller size, lower fat reserves, immature immunological system and inexperience^61^. Furthermore, resource availability affects individual growth, with low resource levels constraining growth rates^7,9^. Under this scenario, individuals are forced to grow over an extended period to attain a size large enough for successful reproduction^12,13^, which leads to a delay in maturity^7^. This suggests that low resource availability played an important role in the dwarfing process of *H. gromovae*, triggering a decrease in growth rate and an associated delay in maturity. Hence, we interpret the small size of the later maturing *H. gromovae* as a consequence of low growth rates and a high juvenile mortality regime due to low resource availability. The analysis of the growth strategies indicates that dwarfing in the two small hipparionins resulted from different adaptive shifts in their life histories under distinct selective pressures.

Extant equids are less diverse in size, form and ecology than they used to be in the past^73^. Nowadays, only domestic horse breeds show a large size range, while size differences in wild taxa are much less pronounced (e.g. *Equus hemionus* weighting 230 kg^74^ and *Equus grevyi* 384 kg^74^). Despite the size disparity, different-sized horse breeds have comparable growth patterns^75^. Gestation lengths also show little variation between breeds^76^, and the time of growth plates closure is similar between small and large horses^77^. Considering this, the size difference in domestic horses is not caused by variations in the duration of growth, but by the higher or lower prenatal^76^ and postnatal^77^ growth rates. Accordingly, we identified a trend indicating higher growth rates in femora of larger extant equids compared to smaller representatives of *Equus*. Hence, differences in growth rates lead to size diversity in extant equids. Similar to the case of *H. gromovae*, epiphyseal closure in the small Iceland horse is delayed, and even later compared to that in Thoroughbreds^77,78^. The lower growth rates and delayed growth plate closure in the small Iceland horse have been related to the original harsher living conditions in which this breed evolved^78^. Likewise, one of the smallest extant wild equid, *Equus hemionus*, dwells in resource-poor environments as the arid and semi-arid steppes and deserts from Asia^79^. In this case, resource limitation led to reduced growth rates and, thus, to a smaller size at maturity. The body size differences between extant taxa, hence, are likely the result of differences in somatic growth rates triggered by differential selective pressures (e.g. resource availability).

Our different life history deductions for the small hipparionins are supported by the different ecological settings inferred for the two bioprovinces. Small ungulates suffer stronger predation pressure than larger ones^80^. They therefore prefer habitats with dense canopy cover (forested areas) that provide shelter and that are more secure than open areas^80–84^, albeit the poor forage supply^80–83^. From Vallesian through Turolian, the Eastern Mediterranean bioprovince was characterised by open dry bushlands and grasslands with low tree cover^85–87^, a risky habitat for small ungulates^80^. The presence of diverse carnivore associations dominated by hyaenids and felids^85^, provides support to our inference from life history models that early maturity and a corollary size decrease in the *macedonicum* morphotype was triggered by high predation pressure. In contrast, the Western Mediterranean biomes during the late Turolian were composed of deciduous forests and xeric woodlands with shrublands and grasslands^88–90^. At the *H. gromovae* site – Rambla de Valdecebro II (=El Arquillo) –, the climate was characterised by a high aridity regime^91^. There was a high carnivore diversity in this area at that time^92,93^, but within a patchy landscape there were forest and xeric woodland areas available for cover. These more forested areas could have provided site humidity and increased protection against predation^82,83^, habitat parameters preferred by extant small ungulates^80^. This suggests that *H. gromovae*, and possibly also the sympatric smaller *Hipparion periafricanum*, likely dwelled in such forested and more secure habitats. Although conferring the advantage of protection, closed areas support fewer grasses and forbs compared to open habitats because of the shading effect of the canopy^83^. The quantity of available forage, hence, is lower under denser tree cover^82,83,94^. Additionally, these small hipparions must have faced longer periods of resource stress because of the high aridity of El Arquillo site^91^, which most likely affected the quality and quantity of available resources^71^. Such adverse climatic conditions must have been an additional problem for equids because their digestive strategy of hindgut fermentation makes them dependent on continuous resource supply^95^. The environmental scenario of low and fluctuating resource levels provides support to our inference from life history models that *H. gromovae* grew at low rates and delayed maturity, which led to a reduction of adult body size. It is conceivable that these resource conditions might have also affected juvenile mortality as observed in extant ungulate populations^59,66–70^.

The causes and mechanisms behind size decrease trends on continents are not completely understood. In this survey focused on the bone histology of different-sized hipparionins, we tested if changes in life history traits are related to size decrease in continental settings. We recognised opposed growth strategies in the two dwarfed lineages, a faster life history characterised by an early maturity in the Greek small hipparion vs. a slower strategy with slow growth and later maturity in the Spanish one; otherwise leading to parallel size shrinking under different ecological scenarios. Based on Palkovacs^7^ life history model, we consider the size decrease as a by-product of life adaptations to differential ecological constraints. Specifically, we interpret the shift of the Spanish small *H. gromovae* towards a slow life history as a response to limited and unpredictable resource supply and an associated increase in juvenile mortality, and the shift of the Greek small *macedonicum* morphotype towards a fast life history as an adaptation to cope with increased adult mortality regimes. Our results show that there is more than one possible life history strategy behind evolutionary dwarfing, and that bone histology is a powerful tool to unravel the mechanisms involved.

## Material and Methods

Previous studies of the characterization and variability of equid bone tissue^43,44^ have provided evidences of the usefulness of metapodial bone microstructure for life history reconstruction in this group. Moreover, we used metapodials due to their higher abundance in fossil assemblages and the possibility to assign them to a specific morphotype. We sectioned 31 hipparionin metapodials from the late Miocene of Greece and Spain for histological analysis. The Greek sample comprises metacarpals and metatarsals of one small (*macedonicum* morphotype) and two large (*primigenium* and *dietrichi* morphotypes *sensu* Vlachou^35^) hipparionin morphotypes from seven fossil sites ranging from early Vallesian to late Turolian (Table 2). Large-sized hipparions are used for comparative purposes. The Spanish sample includes metapodials from one small species (*Hipparion gromovae*) and one specimen of the large *H. truyolsi*, both from a late Turolian fossil site of the Teruel Basin (Table 2). Metapodial histology of other large-to-medium sized hipparionins from early Vallesian and middle Turolian of Spain have already been studied in an exhaustive survey by Martinez-Maza *et al*.^44^.

**Table 2:** Sample analysed and results of body mass estimations.

	Group	Body Mass estimation (kg)	Fossil Site and Age (MN)	Element	Specimen	Collection	LM mid-shaft Diameter (mm)
Eastern Mediterranean	*primigenium* morphotype	**188.92** (±43.35 SD, N = 99)	Pentalophos (MN9)	Metacarpal	PNT-22	LGPUT	26.17
			Pentalophos (MN9)	Metatarsal	PNT-4	LGPUT	25.81
			Ravin de la Pluie (MN10)	Metacarpal	RPL-nn	LGPUT	28.53
			Ravin de la Pluie (MN10)	Metatarsal	RPL-3	LGPUT	27.05
			Nikiti-1 (MN10)	Metacarpal	NKT-22	LGPUT	27.75
	*dietrichi* morphotype	**143.16** (±28.69 SD, N = 279)	Nikiti-2 (MN11)	Metacarpal	NIK-1736	LGPUT	21.92
			Perivolaki (MN12)	Metacarpal	PER-X	LGPUT	21.99
			Perivolaki (MN12)	Metatarsal	PER-1211	LGPUT	22.76
			Perivolaki (MN12)	Metatarsal	PER-342	LGPUT	25.21
			Dytiko-1 (MN13)	Metacarpal	DTK-58	LGPUT	24.42
			Dytiko-1 (MN13)	Metatarsal	DTK-149	LGPUT	24.92
			Dytiko-1 (MN13)	Metatarsal	DTK-106	LGPUT	27.01
			Dytiko-1 (MN13)	Metatarsal	DTK-104	LGPUT	26.34
	*macedonicum* morphotype	**76.09** (±15.04 SD, N = 258)	Ravin de la Pluie (MN10)	Metacarpal	RPL-44	LGPUT	21.51
			Nikiti-1 (MN10)	Metatarsal	NKT-nn	LGPUT	20.33
			Nikiti-2 (MN11)	Metacarpal	NIK-1698	LGPUT	19.85
			Nikiti-2 (MN11)	Metacarpal	NIK-nn	LGPUT	18.65
			Vathylakos-2 (MN11)	Metatarsal	VAT-112	LGPUT	19.71
			Perivolaki (MN12)	Metacarpal	PER-23	LGPUT	20.43
			Perivolaki (MN12)	Metacarpal	PER-425	LGPUT	20.49
			Perivolaki (MN12)	Metatarsal	PER-380	LGPUT	20.50
			Perivolaki (MN12)	Metatarsal	PER-485	LGPUT	20.29
Western Mediterranean	*Hipparion gromovae*	**84.33** (±12.86 SD, N = 80)	Rambla de Valdecebro II (MN13)	Metacarpal	IPS-96274	ICP	21.91
			Rambla de Valdecebro II (MN13)	Metacarpal	IPS-96275	ICP	19.02
			Rambla de Valdecebro II (MN13)	Metacarpal	IPS-101807	ICP	21.12
			Rambla de Valdecebro II (MN13)	Metacarpal	IPS-101810	ICP	20.43
			Rambla de Valdecebro II (MN13)	Metatarsal	IPS-96276	ICP	20.92
			Rambla de Valdecebro II (MN13)	Metatarsal	IPS-96277	ICP	21.51
			Rambla de Valdecebro II (MN13)	Metatarsal	IPS-101808	ICP	22.22
			Rambla de Valdecebro II (MN13)	Metatarsal	IPS-01809	ICP	22.81
	*H. truyolsi*	**192.46** (±17.37 SD, N = 8)	Rambla de Valdecebro II (MN13)	Metatarsal	IPS-28842	ICP	28.73

Lateromedial diameter (LM) at mid-shaft is provided as a metapodial size proxy. LGPUT: Material sampled from the collections of the Laboratory of Geology and Paleontology at the University of Thessaloniki; ICP: Material sampled from the collections of the Institut Català de Paleontologia Miquel Crusafont.

To support our interpretations of the growth rates in hipparionins, we also analysed bone appositional rates in extant equids of different body sizes. Because the femur develops over a longer time period^96^, its use in *Equu*s bone histology is preferred if possible^43^. Hence, we calculated the appositional growth in femora to estimate the growth rates of different-sized extant equids. The entire extant sample was composed by eight femora of wild and domestic equids; two belonging to *Equus grevyi*, two to *Equus hemionus*, and four to different-sized horse breeds (Iceland, Welsh, Hackney and Shetland) (Supplementary Table S1).

Adult body mass estimations of the fossil Greek morphotypes were calculated from the material published in Vlachou^35^ and were kindly provided by the author. Body mass estimations of the Spanish sample were calculated (Supplementary Table S2) following the same measurements and regressions used for the Greek metapodials (Table 2). These estimations were obtained for each morphotype from measurements on metapodials (Mc10, Mt10, Mc13, Mt13, following Eisenmann *et al*.^97^) and using the equations provided by Eisenmann and Sondaar^98^ and Scott^53^.

### Preparation of histological slides

Histological slides were produced following the standard protocol of our laboratory^43,99^. Mid-shaft blocks of each metapodial were embedded in epoxy resin (Araldite 2020) and sectioned using an IsoMet low-speed saw (Buehler). The exposed surfaces were polished using a grinder polisher (Buehler, MetaServ 250) and glued to a glass slide using the same epoxy resin. The mounted samples were cut using a diamond saw (Buehler, Petrothin) up to a thickness of 300 μm and grounded to 150–100 μm, using the grinder polisher. Finally, the slides were dehydrated in alcohol gradients and immersed in a histological clearing agent (Histo-Clear II) prior to cover them with a DPX medium.

The histological samples were studied under polarised light using a Zeiss Scope.A1 microscope with an attached digital camera (AxioCam ICc5). The slides were examined using a retardation filter of ¼ λ to improve the observation of the bone tissues and growth marks^100^. The micrographs of the cortex were merged using Adobe Photoshop^®^ and analysed with Image J software.

### Bone histology

We analysed bone tissues and bone growth marks to reconstruct metapodial growth. Description of bone tissues types follows the classification of Francillon-Viellot *et al*.^101^ and de Margerie *et al*.^48^. The analysis of cyclical growth marks (CGM) of annual periodicity^99^ provide the temporal basis for the reconstruction of metapodial growth and the assessment of certain life history traits^6,37,38^. Where the growth marks were faint due to poor tissue preservation or partially eroded by secondary osteons, we retrocalculated their track by superimposition^102^. The identification of a non-cyclical growth mark, the neonatal line^47^, in the innermost cortical area of almost all metapodials allowed the estimation of bone size at birth.

The significant decrease in bone growth rate marks the attainment of bone maturity. This event is identified by the periosteal deposition of slow-growing lamellar bone after fast-growing fibrous tissue^37^ and by the narrowing of consecutive growth zones^51^. By counting the CGM that precede the deposition of the avascular lamellar bone found in the outermost cortex of bone (external fundamental system (EFS) *sensu* Woodward *et al*.^102^), some authors have inferred the organism’s age at sexual maturity^38,103^ or skeletal maturity^44,57^ from mainly tibia and femora tissues. Nacarino-Meneses *et al*.^43^, however, provided evidence that the decrease in periosteal growth rate in equids metapodials – represented by the inflection point of the growth curves – is an indicator of their epiphyseal fusion and the end of longitudinal growth. As metapodials fuse their epiphyses earlier than other long bones^96^, the identification of the moment of metapodial growth decrease only provides a relative age at skeletal maturity. Thus, we obtain a proxy of maturity attainment, instead of an absolute age at which the animal should end its growth. We used this ‘*relative maturity*’ to compare between taxa and to identify which species tends to delay or to advance it.

In the extant femur sample used for comparative purpose, we estimated the growth rate between the first and the second year of growth. We identified these two CGM and measured their perimeter. The estimation of the growth rate was calculated as the perimeter difference between the second and the first CGM.

### Growth curves

Growth data in metapodials were obtained by counting the bone growth marks and by calculating their perimeters. Contrary to studies on dinosaurs^41,104^ that use body mass estimations for each growth cycle, our growth curves are based on direct measures. We did not estimate body mass at each growth cycle because the correlation between equid metapodial mid-shaft dimensions and body mass is not significant^25^, and the regressions are based adult individuals. Instead, we took the direct skeletal measurement of the mid-shaft circumference as a proxy of body size, a more conservative alternative to an estimate of body mass^52^. In those cases where exact mid-shaft cuts were not possible due to the fragmentary nature of the specimen (IPS-101809, *H. gromovae* metatarsal), the bone circumference is slightly overestimated. This is particularly true in metatarsals which are anteroposteriorly wider in more proximal planes; however, the growth trajectory can be equally reconstructed. From the available sample, we used only those specimens for the growth curve reconstructions where the radial growth was finished (18 metapodials). The other specimens have been analysed for bone tissue characterization, growth mark superimposition, and the study of the ontogenetic development.

Similar to other biological processes, growth is generally modelled using nonlinear sigmoidal equations^105,106^. Among several models, and considering the nature of our data, we used the logistic equation (1) ^1,107^ to describe the pattern of metapodial radial growth because it presents good fitting values (Akaike Information Criterion) (Supplementary Table S3) and provides the most realistic asymptotic size values. A similar logistic model has been used to describe the circumferential growth of dinosaurian femora and tibiae^40,108^, as well as dinosaur body mass growth^104^. We used the nonlinear least squares fitting of the PAST software to fit the curves equations to our data^109^.

1 $$y(t)=\frac{A}{(1+b{e}^{-kt})}$$

In sigmoidal equations, the parameter *A* represents the final asymptotic value to which the response variable approaches in an exponentially decreasing rate after reaching the growth inflection point^50^. In our case, the *A* parameter shows the final adult metapodial circumferential size. The mean relative growth rate, or the relative velocity at which the response variable is approaching to this final size, is characterised by the exponent *k*^108^, while the parameter *b* represents the quotient between the initial and final size^110^. Hence, the parameter *k* expresses the ratio of the maximum growth rate in relation to the adult size, indicating delayed maturity when *k* is low and advanced maturity when *k* is high^111^. These growth curve parameters were calculated for each specimen and the results were also averaged to obtain a mean growth curve for the metacarpals and metatarsals of each group^42^. The rate at which bone grows was calculated from the derivative (2) of this logistic equation (1), representing the instantaneous growth rate at a concrete time. It should be considered that the calculated growth rate values expressed as mm/year do not represent a real approximation of the rate at which those bones were growing, because bone growth rate varies with the season of the year^99^. However, these values represent useful estimates for comparison^42^. In order to compare the growth rates between species, we estimated the relative growth rate (RGR) extracting the effect of size (3), because growth rates tightly scale with size^1^. The relative growth rate is defined as the increase per unit of time relative to the size at a concrete point^112^.

2 $$GR(t)=\frac{{\rm{\partial }}y}{{\rm{\partial }}t}=\frac{\begin{array}{c}A\,b\,k\,{e}^{kt}\end{array}}{\begin{array}{c}{(b+{e}^{kt})}^{2}\end{array}}$$

3 $$RGR(t)=\frac{1}{y(t)}\,\frac{{\rm{\partial }}y}{{\rm{\partial }}t}=\frac{\begin{array}{c}A\,b\,k\,{e}^{kt}\end{array}}{\begin{array}{c}y(t){(b+{e}^{kt})}^{2}\end{array}}$$

Additionally, we calculated the time required to attain the 95% of the final size (4) as an indicator of the growth duration time^108^.

4 $${t}_{95A}=-\frac{1}{k}{\rm{l}}{\rm{n}}(\frac{0.05A}{0.95A\,b})$$

### Statistics

Statistical analyses and graphs were performed using Past 3.14^109^ and Microsoft Office Excel. Pearson’s correlation was used for the linear correlation analysis. A significance level of 0.05 was used for all tests.

## Electronic supplementary material

Supplementary Figures and Tables

## Data Availability

The data sets generated and/or analysed during the current study are included on this published article. Raw data not included is available from the corresponding author on reasonable request.
