# Publisher Correction: Bone histology provides insights into the life history mechanisms underlying dwarfing in hipparionins

**DOI:** 10.1038/s41598-019-45207-x

**Published:** 2019-07-11

**Authors:** Guillem Orlandi-Oliveras, Carmen Nacarino-Meneses, George D. Koufos, Meike Köhler

**Affiliations:** 1 Institut Català de Paleontologia Miquel Crusafont (ICP), Campus de la Universitat Autònoma de Barcelona, 08193 Bellaterra Barcelona, Spain; 2 Department of Geology, Laboratory of Geology and Palaeontology, Aristotle University of Thessaloniki, 54124 Thessaloniki, Greece; 3 ICREA, Pg. Lluís Companys 23, 08010 Barcelona, Spain

Correction to: *Scientific Reports* 10.1038/s41598-018-35347-x, published online 21 November 2018

The original version of this Article contained errors.

Due to a typesetting error, Table 1 contained incorrect values in the ‘t95A’, ‘GR (t = 0.5)’, ‘RGR (t = 0.5)’, ‘GR (t = 1)’, ‘RGR (t = 1)’, ‘GR (t = 2)’, ‘RGR (t = 2)’, ‘GR (t = 3)’, and ‘RGR (t = 3)’ columns.

**Table 1:** Logistic growth curve parameters and growth estimates for each adult metapodial.

GROUP	BONE	CODE	A (mm)	k (year^−1^)	t95A (year)	GR (t = 0.5) (mm/year)	RGR (t = 0.5)(year^-1^)	GR (t = 1) (mm/year)	RGR (t = 1)(year^−1^)	GR (t = 2) (mm/year)	RGR (t = 2)(year^−1^)	GR (t = 3) (mm/year)	RGR (t = 3) (year^−1^)
*primigenium* morphotype	MC	NKT-22	81.54	0.66	2.46	7.35	0.108	5.79	0.081	3.36	0.044	1.85	0.023
	MC	RPL-nn	83.74	1.02	1.81	11.89	0.170	8.20	0.110	3.41	0.043	1.30	0.016
	MT	PNT-4	86.53	0.99	2.00	13.07	0.186	9.29	0.123	4.07	0.049	1.61	0.019
	**Mean**	***pg*** **MC**	**82.64**	**0.84**	**2.07**	**9.60**	**0.139**	**7.08**	**0.097**	**3.49**	**0.045**	**1.60**	**0.020**
		***pg*** **MT**	**86.53**	**0.99**	**2.00**	**13.07**	**0.186**	**9.29**	**0.123**	**4.07**	**0.049**	**1.61**	**0.019**
*macedonicum* morphotype	MC	RPL-44	69.06	0.86	1.94	7.75	0.133	5.63	0.091	2.69	0.041	1.20	0.018
	MC	PER-23	65.57	1.27	1.42	10.31	0.184	6.30	0.105	2.00	0.031	0.59	0.009
	MC	PER-425	64.47	0.88	1.83	6.99	0.127	5.01	0.086	2.35	0.038	1.03	0.016
	MT	VAT-112	64.60	1.88	0.90	10.91	0.188	4.83	0.078	0.79	0.012	0.12	0.002
	MT	PER-485	70.71	1.32	1.16	9.32	0.149	5.38	0.081	1.57	0.023	0.43	0.006
	**Mean**	***mc*** **MC**	**66.37**	**1.00**	**1.69**	**8.40**	**0.149**	**5.74**	**0.096**	**2.39**	**0.037**	**0.92**	**0.014**
		***mc*** **MT**	**67.66**	**1.60**	**1.01**	**10.28**	**0.170**	**5.21**	**0.081**	**1.14**	**0.017**	**0.23**	**0.003**
*dietrichi* morphotype	MC	PER-X	78.02	0.65	3.05	8.59	0.141	7.03	0.108	4.33	0.061	2.48	0.034
	MC	DTK-58	76.29	0.85	2.14	9.34	0.148	6.92	0.103	3.42	0.048	1.56	0.021
	MT	PER-1211	82.96	0.94	2.16	12.46	0.188	9.11	0.127	4.23	0.054	1.77	0.022
	MT	PER-342	86.29	1.04	2.02	14.63	0.213	10.32	0.138	4.35	0.053	1.64	0.019
	MT	DTK-106	87.97	1.02	1.97	13.80	0.194	9.71	0.126	4.13	0.049	1.59	0.018
	**Mean**	***dt*** **MC**	**77.15**	**0.75**	**2.54**	**9.08**	**0.146**	**7.08**	**0.107**	**3.92**	**0.055**	**2.00**	**0.027**
		***dt*** **MT**	**85.74**	**1.00**	**2.04**	**13.63**	**0.198**	**9.72**	**0.130**	**4.24**	**0.052**	**1.67**	**0.020**
*H. gromovae*	MC	IPS-101807	65.53	0.79	2.25	7.42	0.137	5.62	0.098	2.93	0.048	1.42	0.022
	MC	IPS-96274	63.83	0.77	2.29	6.93	0.131	5.29	0.095	2.82	0.047	1.40	0.023
	MT	IPS-101809	81.00	0.95	2.15	12.34	0.191	9.00	0.129	4.15	0.054	1.73	0.022
	MT	IPS-96276	66.09	1.01	1.92	9.94	0.184	6.96	0.119	2.96	0.047	1.14	0.018
	**Mean**	***gr*** **MC**	**64.68**	**0.78**	**2.27**	**7.17**	**0.134**	**5.45**	**0.096**	**2.88**	**0.047**	**1.41**	**0.023**
		***gr*** **MT**	**73.55**	**0.98**	**2.04**	**11.15**	**0.188**	**7.97**	**0.124**	**3.53**	**0.051**	**1.42**	**0.020**
*H. truyolsi*	MT	IPS-28842	97.00	1.30	1.39	15.41	0.185	9.27	0.104	2.85	0.030	0.80	0.008
	**Mean**	***ty*** **MT**	**97.00**	**1.30**	**1.39**	**15.41**	**0.185**	**9.27**	**0.104**	**2.85**	**0.030**	**0.80**	**0.008**

Mean growth curve parameters and estimates for the metacarpals (MC) and metatarsals (MT) of each group are also provided. *A:* asymptotic circumferential metapodial size; *k*: mean relative velocity; *t95A*: time required to attain the 95% of the final size. Growth rates (GR) and relative growth rates (RGR) at different points of the metapodial growth are also shown.

In addition, in Table 2 the headings “Western Mediterranean” and “Eastern Mediterranean” were reversed.

These errors have now been corrected in the PDF and HTML versions of the Article.

