# Supplementary material for: Bone histology provides insights into the life history mechanisms underlying dwarfing in hipparionins
Source: Sci Rep. 2018 Nov 21;8:17203. doi: 10.1038/s41598-018-35347-x (PMC6249282; doi:10.1038/s41598-018-35347-x)
Supplement: Supplementary file 1 — Supplementary Figures and Tables [file 41598_2018_35347_MOESM1_ESM.pdf]

Supplementary Material for:

Bone histology provides insights into the life history mechanisms underlying dwarfing in hipparionins.

Guillem Orlandi-Oliveras, Carmen Nacarino-Meneses, George D. Koufos & Meike Köhler

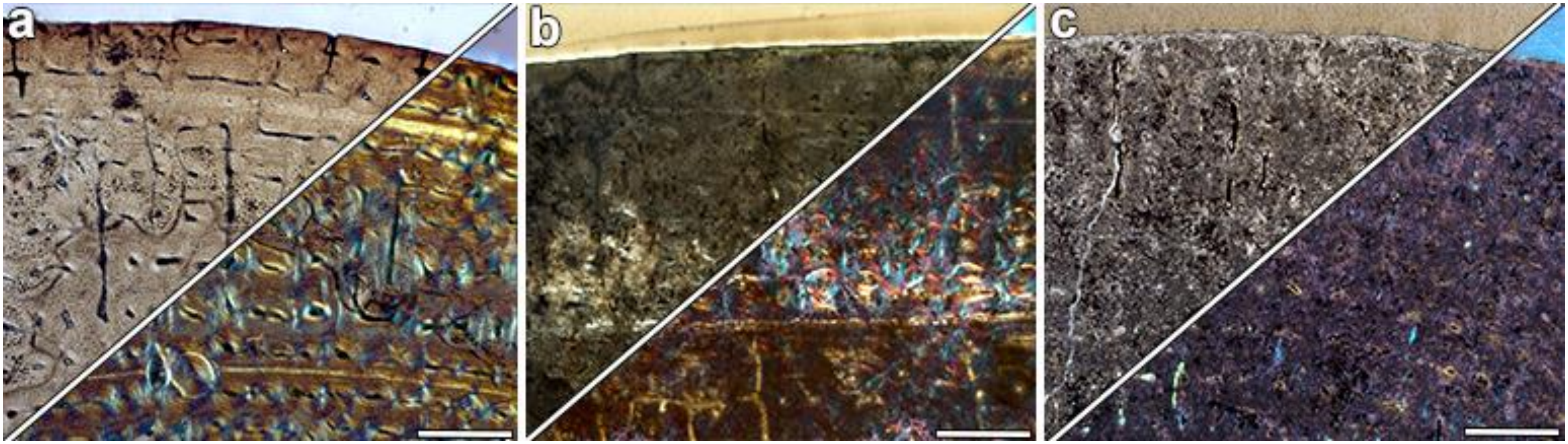

Supplementary Figure S1. Histological preservation of the sample. Top half part of the microphotographs shows bone cross-sections observed under polarised light, lower half images show bone cross-sections observed under polarised light using a 1/4λ filter. Scale: 0.5 mm. **(a)** Well preserved bone tissue in the metapodials from the Teruel basin (Spain). *H. gromovae* metacarpal IPS-96275. **(b)** Poorly preserved bone tissue where histological structures are still distinguishable in almost all the Greek specimens. *H.* aff. *platygenys* (*dietrichi* morphotype) metatarsal DTK-104. **(c)** Severely damaged microstructure in the samples from Nikiti-2 (NIK) fossil site (Greece). *H. philippus* (*dietrich*i morphotype) metacarpal NIK-nn.

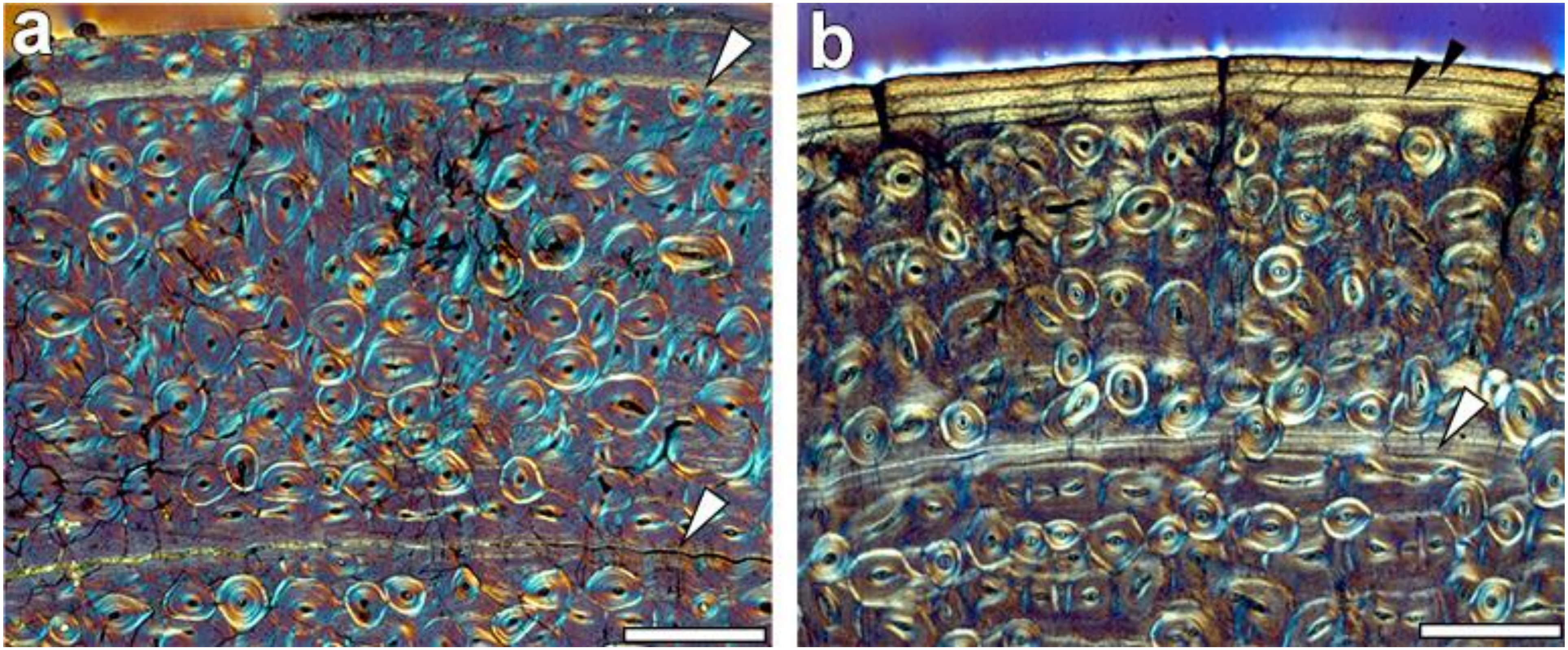

Supplementary Figure S2. Bone cortex with a high density of Haversian Systems, albeit conserving bone growth marks. Images were obtained under polarised light using a 1/4λ filter. Scale: 0.5 mm. **(a)** *H. philippus* (*dietrich*i morphotype) metacarpal PER-X. **(b)** *H. truyolsi* metatarsal IPS-28842.

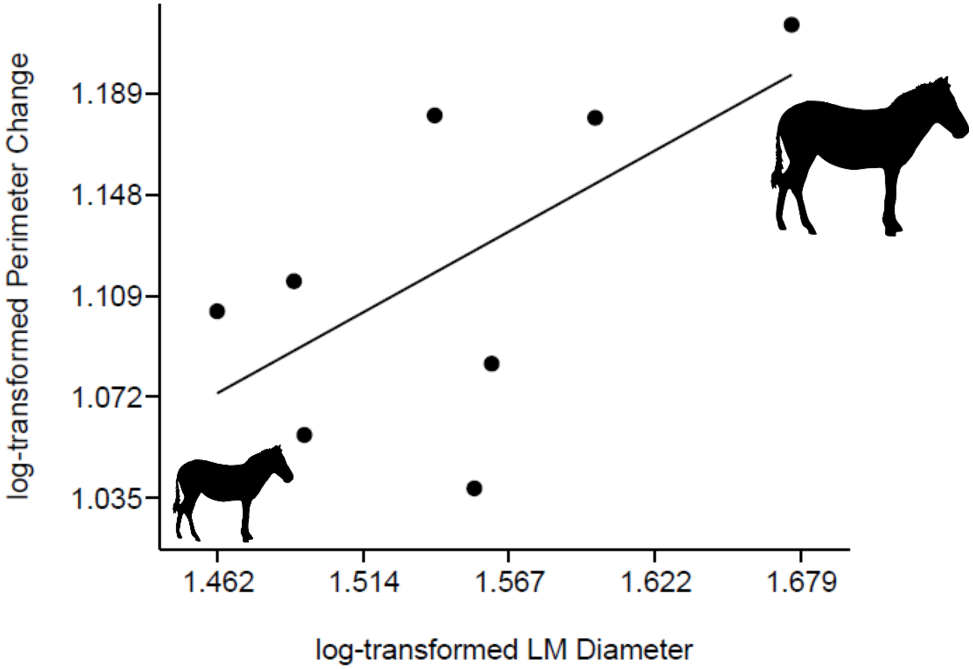

Supplementary Figure S3. Linear regression relating the increase in perimeter between the first and the second cyclical growth mark and the lateromedial (LM) diameter of extant equid femurs. Both variables were log-transformed to linearise the relationship. See main text for statistics information.

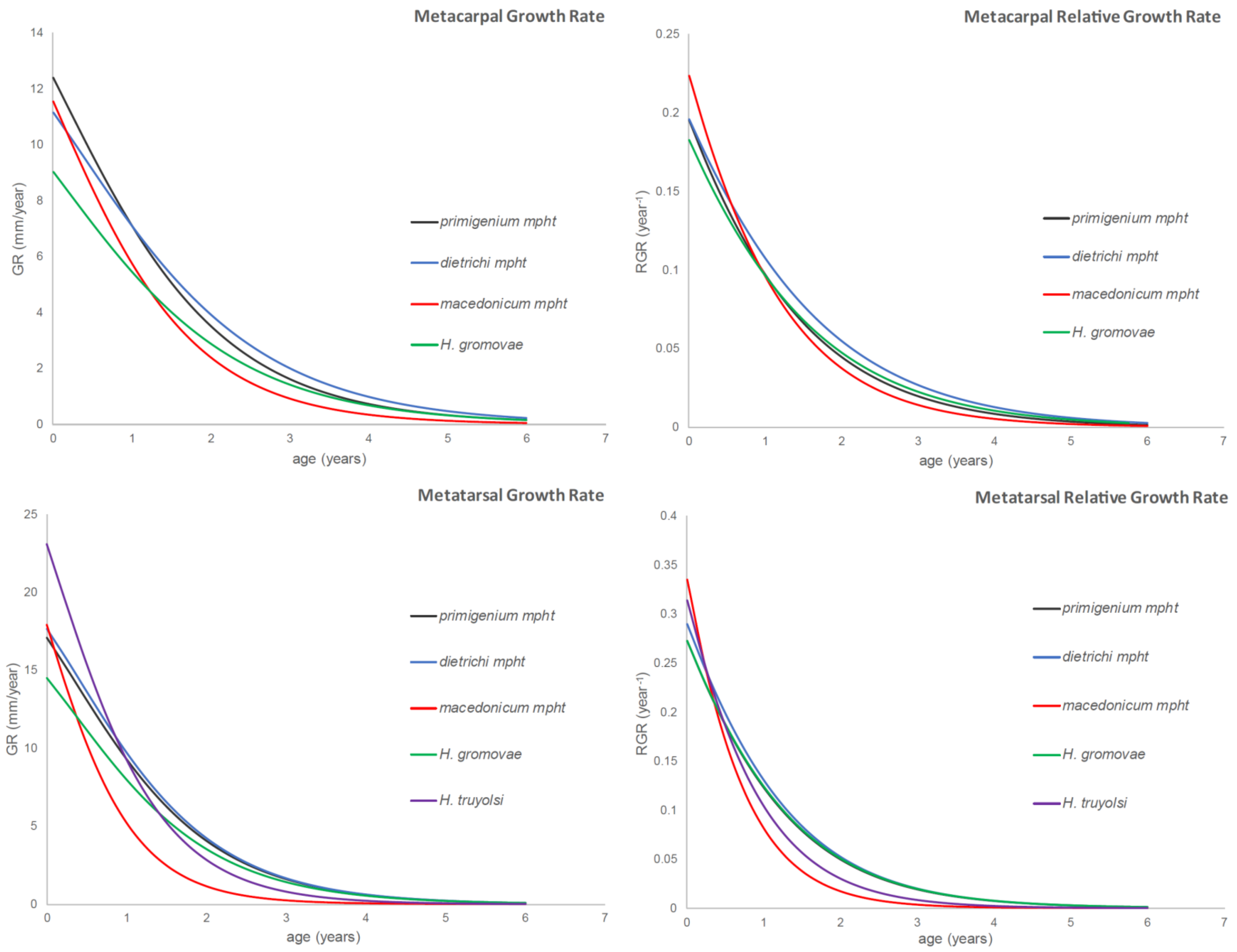

Supplementary Figure S4. Mean growth rate and mean relative growth rate curves for the metacarpals and metatarsals of the hipparionins studied.

Code	Species and Breed	Collection	LM mid-shaft Diameter (mm)	First CGM Perimeter (mm)	Second CGM Perimeter (mm)
IPS-84964	*Equus grevyi*	Zoological Institute of Hamburg University (Hamburg, Germany)	34.63	98.73	113.85
IPS-101804	*Equus grevyi*	Catalan Institute of Paleontology (Barcelona, Spain)	47.35	140.26	156.73
IPS-83876	*Equus hemionus*	Museum of Domesticated Animals (Halle, Germany)	30.83	87.78	100.80
IPS-83877	*Equus hemionus*	Museum of Domesticated Animals (Halle, Germany)	31.09	86.93	98.35
IPS-64287	*Equus caballus* (Iceland)	Zoological Institute and Museum of Kiel University (Kiel, Germany)	39.73	108.76	123.84
IPS-64288	*Equus caballus* (Welsh)	Zoological Institute and Museum of Kiel University (Kiel, Germany)	36.34	105.71	117.81
IPS-64290	*Equus caballus* (Hackney)	Zoological Institute and Museum of Kiel University (Kiel, Germany)	35.81	113.16	124.09
IPS-64291	*Equus caballus* (Shetland)	Zoological Institute and Museum of Kiel University (Kiel, Germany)	28.98	85.78	98.47

Supplementary Table S1. Femurs of the extant equids analysed. Lateromedial diameter (LM) at mid-shaft is provided as a proxy of size. The estimation of the growth rate has been calculated as the difference between the second and the first cyclical growth mark (CGM).

					Scott (1990) Mc10		Eisenmann & Sondaar (1998) Mc10/Mt10		Eisenmann & Sondaar (1998) Mc13/Mt13		Eisenmann & Sondaar (1998) Mc/t10*Mc/t13	
IPS Code	Bone	Species	Mc10 (mm)	Mc13 (mm)	logBM	BM (kg)	lnBM	BM (kg)	lnBM	BM (kg)	lnBM	BM (kg)
28808-1	Metacarpal	*H. gromovae*	27.24	17.50	1.88	76.45	4.18	65.52	4.48	87.86	4.32	75.06
28808-2	Metacarpal	*H. gromovae*	29.20	19.64	1.97	92.29	4.39	80.77	4.78	119.49	4.58	97.85
28808-6	Metacarpal	*H. gromovae*	28.20	18.67	1.92	83.97	4.29	72.73	4.65	104.40	4.46	86.56
28808-8	Metacarpal	*H. gromovae*	27.31	18.44	1.89	76.91	4.19	65.96	4.62	101.01	4.40	81.17
28808-9	Metacarpal	*H. gromovae*	27.01	16.4	1.87	74.64	4.16	63.80	4.30	73.90	4.21	67.53
28808-10	Metacarpal	*H. gromovae*	27.88	18.55	1.91	81.42	4.25	70.27	4.63	102.62	4.44	84.37
29015-7	Metacarpal	*H. gromovae*	27.65	18.35	1.90	79.61	4.23	68.54	4.60	99.70	4.41	82.09
29015-2	Metacarpal	*H. gromovae*	26.10	17.05	1.83	68.10	4.05	57.61	4.41	81.97	4.22	68.01
29015-3	Metacarpal	*H. gromovae*	27.95	-	1.91	81.97	4.26	70.80	-	-	-	-
29015-4	Metacarpal	*H. gromovae*	28.68	19.90	1.94	87.90	4.34	76.52	4.82	123.75	4.50	90.38
29015-5	Metacarpal	*H. gromovae*	-	18.92	-	-	-	-	4.68	108.17	-	-
IPS Code	Bone	Species	Mt10 (mm)	Mt13 (mm)	logBM	BM (kg)	lnBM	BM (kg)	lnBM	BM (kg)	lnBM	BM (kg)
29015-IV	Metatarsal	*H. gromovae*	25.95	17.30	1.89	78.57	4.22	67.68	4.29	72.62	4.23	68.53
29015-V	Metatarsal	*H. gromovae*	27.07	18.80	1.94	87.05	4.33	75.65	4.54	93.97	4.41	82.12
29015-VI	Metatarsal	*H. gromovae*	27.50	17.82	1.96	90.44	4.37	78.85	4.38	79.60	4.35	77.76
29015-VII	Metatarsal	*H. gromovae*	28.94	18.80	2.01	102.35	4.50	90.20	4.54	93.97	4.51	90.43
29015-VIII	Metatarsal	*H. gromovae*	28.63	-	1.99	99.71	4.47	87.68	-	-	-	-
29015-IX	Metatarsal	*H. gromovae*	-	17.33	-	-	-	-	4.29	73.01	-	-
38842-7	Metatarsal	*H. gromovae*	27.38	18.51	1.95	89.48	4.36	77.95	4.49	89.40	4.40	81.57
38842-9	Metatarsal	*H. gromovae*	27.65	19.20	1.96	91.64	4.38	79.99	4.61	100.31	4.47	87.28
28802-2	Metatarsal	*H. gromovae*	28.88	19.11	2.01	101.84	4.50	89.71	4.59	98.70	4.52	92.24
28002-4	Metatarsal	*H. gromovae*	27.50	19.05	1.96	90.44	4.37	78.85	4.58	97.90	4.45	85.63
28002-5	Metatarsal	*H. gromovae*	29.05	18.75	2.01	103.30	4.51	91.10	4.54	93.20	4.51	90.58
28002-12	Metatarsal	*H. gromovae*	25.88	17.09	1.89	78.06	4.21	67.20	4.25	69.92	4.21	67.07
28837	Metatarsal	*H. truyolsi*	37.90	24.21	2.29	196.85	5.21	183.55	5.33	205.55	5.26	192.12
28842	Metatarsal	*H. truyolsi*	36.55	24.90	2.26	180.27	5.12	166.82	5.41	224.55	5.25	189.99

Supplementary Table S2. Measurements of metacarpals and metatarsals of *Hipparion gromovae* and *Hipparion truyolsi* from Rambla de Valdecebro II (= El Arquillo) fossil site used for the body mass estimations. See Methods for information about the body mass estimations.

	Measure: Diaphyseal Perimeter		
**Model** / **Specimen**	**von Bertalanffy AIC**	**Logistic AIC**	**Gompertz AIC**
NKT-22	30.502	30.540	30.520
RPL-nn	30.439	30.228	30.321
RPL-44	19.040	18.700	18.887
PER-23	30.070	30.470	30.560
PER-425	20.920	20.468	20.690
PER-X	37.012	36.097	36.547
DTK-58	NA	NA	NA
101807	18.227	18.110	18.160
96274_2	14.073	14.124	14.088
PNT-4	31.010	30.523	30.747
VAT-112	NA	NA	NA
PER-485	30.004	30.015	30.008
PER-1211	19.092	18.451	18.730
PER-342	33.156	32.046	32.574
28842	30.218	30.090	30.143
96276	32.524	33.085	32.791
DTK-106	30.317	30.104	30.187
101809	30.148	30.048	30.068

Supplementary Table S3. Akaike Information Criterion (AIC) for the curve fitting of three different equation growth models (von Bertalanffy. Logistic. Gompertz) in 18 adult hipparionine metapodials. The model exhibiting lower AIC values has been shadowed.
